# Association between Medical Avoidance Behavior and Lifestyle Changes during the Early Phase of Coronavirus Disease 2019 Emergency in Tokyo: A Cross-Sectional Study in a Gastric Cancer Screening Cohort

**DOI:** 10.31662/jmaj.2024-0226

**Published:** 2025-09-26

**Authors:** Natsumi Terada, Ayami Ono, Shiori Tanaka, Tetsuji Minami, Fuyuki Yamada, Toshiki Yamashita, Sawori Osada, Hitoshi Mio, Masahiko Nakamura, Masakatsu Uchihara, Nakayuki Yoshimura, Masao Yano, Yasuhiko Nagata, Mikio Masuda, Ryohei Itagaki, Toru Kakuta, Akira Torii, Manami Inoue

**Affiliations:** 1 Division of Prevention, National Cancer Center Institute for Cancer Control, Tokyo, Japan; 2 Division of Cohort Research, National Cancer Center Institute for Cancer Control, Tokyo, Japan; 3 Adachi-ku Medical Association, Adachi City, Tokyo, Japan; 4 Katsushika Medical Association, Katsushika City, Tokyo, Japan; 5 Minato-ku Medical Association, Minato City, Tokyo, Japan; 6 Mitaka city Medical Association, Mitaka City, Tokyo, Japan; 7 Machida city Medical Association, Machida City, Tokyo, Japan; 8 Nishitokyo Medical Association, Nishitokyo City, Tokyo, Japan; 9 Tokyo Kita-ku Medical Association, Kita City, Tokyo, Japan; 10 Tokyo Medical Association, Chiyoda City, Tokyo, Japan

**Keywords:** COVID-19, questionnaire survey, patient behavior, Japan

## Abstract

**Introduction:**

This study examines patient behavior in Tokyo, Japan, during the early phase of the coronavirus disease 2019 (COVID-19) pandemic.

**Methods:**

As part of a prospective cohort study on gastric cancer screening participants, we conducted a cross-sectional survey from April 2021 to March 2022. The survey included 1,554 participants (40.5% men, median age: 62, age range: 32-92). Specifically, we investigated whether participants continued hospital follow-up visits and underwent medical checkups and disease prevention screenings during the initial state of emergency in Japan (April to May 2020). We also explored changes in daily life and the psychological effects of COVID-19.

**Results:**

During the state of emergency, 12.7% of men and 20.4% of women discontinued follow-up visits, with the primary reason being a desire to avoid human contact. Additionally, 6.5% of men and 14.2% of women ceased medical checkups and screenings. Among women, those who reported increased time spent on housework and family care, or experienced heightened stress and conflicts with housemates, were significantly more likely to discontinue or delay follow-up visits or treatment (odds ratio [OR] 1.55 [1.08-2.23], OR 1.53 [1.06-2.21]).

**Conclusions:**

Our findings indicate that some middle-aged and elderly Japanese residents in urban areas avoided healthcare services to reduce the risk of COVID-19 infection. A subset of individuals continued this behavior even after the pandemic subsided. It is crucial to prioritize regular checkups for managing chronic illnesses and preventing new conditions. Effective communication strategies should be developed in collaboration with national and local governments.

## Introduction

Coronavirus disease 2019 (COVID-19) has significantly impacted lives worldwide. In Japan, the first COVID-19 case was reported on January 14, 2020, and the first wave of the epidemic occurred in April 2020 ^(1)^. The Japanese government declared a state of emergency (SOE) for seven prefectures from April to May 2020, including Tokyo ^(2)^. During the SOE, Tokyo residents were asked to stay at home whenever possible; however, exceptions were made for essential activities such as attending medical facilities, purchasing food, going to work, and other necessary tasks ^(3)^.

Although there were no restrictions on visiting healthcare facilities, there was concern that patients might avoid seeking medical care under these exceptional circumstances. Reports from Japanese individuals receiving medical treatment indicated a decrease in the number of outpatient visits during the COVID-19 pandemic ^(4), (5), (6)^. However, to our knowledge, no studies have focused on the general population of Tokyo, which had the highest number of COVID-19 infections among Japan’s prefectures. Therefore, we conducted a cross-sectional, paper-based questionnaire survey as part of the ongoing Tokyo Gastric Cancer Screening Study (TGCSS) to examine the behavior of Tokyo residents during the early phase of the COVID-19 pandemic.

## Materials and Methods

### Study population

This survey was conducted as a follow-up to the TGCSS, which took place between April 27, 2021, and March 31, 2022. TGCSS is a prospective cohort study of Tokyo residents who underwent gastric cancer screening, aiming to assess the effectiveness of a risk-stratified screening approach for gastric cancer in Japan. Recruitment occurred from October 2016 to March 2020 at medical institutions and screening sites across seven wards and cities in Tokyo (Minato City, Kita City, Adachi City, Katsushika City, Mitaka City, Nishitokyo City, and Machida City). Annual follow-up surveys are planned for 10 years. The study protocol was approved by the research ethics committees of the National Cancer Center (approval number: 2015-315).

### Procedure and questionnaire design

Questionnaires were distributed alongside the annual follow-up survey from the TGCSS. The survey introduction and instructions, including the purpose, the voluntary nature of participation, and assurance of anonymity, were provided on the first page. Participants who were interested in the survey were asked to give informed consent. A total of 3,060 individuals were sent the questionnaire, of whom 1,681 (54.9%) agreed to participate.

The questionnaire consisted of 18 questions. The primary questions focused on whether participants continued scheduled follow-up visits or treatment at the hospital and whether they underwent medical checkups to prevent disease. One such question asked, “Are you discontinuing or postponing follow-up visits or treatments at the hospital during SOE?” (yes or no). Participants were also asked whether they received medical checkups or screenings during the SOE (e.g., “Decided not to take the checkup,” “Received a scheduled medical checkup”). These checkups, which could be free or low-cost, were provided by municipal governments, employers, or through private comprehensive checkups. Several additional questions explored life changes and psychological effects due to COVID-19. Some asked participants to recall their circumstances during the period the SOE was declared (April 7 to May 25, 2020 ^(2)^), while others asked about current life compared to pre-pandemic conditions.

Participants were surveyed regarding lifestyle changes due to COVID-19, including changes in work (e.g., increased remote work, changes in work hours or wages), housework and family care time, household income, and interactions with others. Participants reported changes in contact frequency with non-cohabitees and household members, the frequency of conflicts and irritability with roommates compared to pre-COVID-19 (rated on a 5-point scale; options included “live alone” and “don’t want to answer”), as well as methods for stress reduction (e.g., exercising, walking, watching TV, drinking ) and infection prevention measures (e.g., wearing a face mask, avoiding eating out, working remotely). Lifestyle factors such as smoking, drinking, body weight, exercise, and eating habits were also assessed.

Mental health was evaluated using validated scales. Loneliness was measured using the University of California, Los Angeles (UCLA) Three-Item Loneliness Scale ^(7), (8)^, specifically the verified Japanese version ^(9)^ translated by Arimoto and Tadaka ^(10)^. Responses were scored from 1 (Rarely) to 3 (Often) and summed, with a score of 6 or higher indicating loneliness. The Kessler 6 scale (K6) ^(11)^, a shortened version of K10 ^(12)^, was used to assess psychological distress through six questions on emotions like nervousness and hopelessness. Responses ranged from 0 (None of the time) to 4 (All of the time), with scores between 5 and 12 indicating mild psychological distress, and scores of 13 or higher signifying severe distress. The Fear of Coronavirus-19 Scale (FCV-19S) ^(13)^ was also used, with a translated version by Tachikawa et al. ^(14)^. Participants rated seven fear-related statements on a 5-point scale from 1 (Not at all) to 5 (Always), and responses were summed (total score range: 5-35) to measure fear levels, although a standardized scoring method has not yet been established.

### Statistical analysis

Of the 1,681 respondents, 1,554 provided their gender and age and were included in the analysis. Analysis of variance and χ^2^ tests were performed to examine the age and regional distribution of participants, as well as those who discontinued or postponed follow-up visits during SOE. Logistic regression analysis was used to compare the association between the refraining from seeing a doctor and the questionnaire responses. Analyses were conducted using a crude model (model 1), a model adjusted for gender, age (category), study area, and employment status (model 2), and stratified by gender. Statistical analyses were carried out using STATA version 17.0 (Stata Corp LLC, College Station, TX, USA), with statistical significance set at p < 0.05.

## Results

The analysis included 1,554 participants (629 men, 40.5%; 925 women, 59.5%). Their age and regional distribution are shown in Table 1. The average age was 60.4 years, and by area, Nishitokyo City had the highest participation rate, with 79.9% of participants being employed.

**Table 1.** Distribution of Participants by Age, Gender, and Study Area.

Participant Characteristics	Overall	Men	Women	p*
	n = 1,554	n = 629	n = 925	
Age, mean (SD)	60.4 (11.5)	63.1 (11.3)	58.6 (11.2)	<0.001^(a)^
Age category, n (%)				
30-39	11 (0.7)	2 (0.3)	9 (1.0)	
40-49	328 (21.1)	95 (15.1)	233 (25.2)	
50-59	360 (23.2)	128 (20.4)	232 (25.1)	
60-69	461 (29.7)	185 (29.4)	276 (29.8)	
70-79	346 (22.3)	189 (30.1)	157 (17.0)	
80+	48 (3.1)	30 (4.8)	18 (2.0)	
Study area, n (%)				0.06^(b)^
Nishitokyo City	668 (43.0)	66 (45.5)	382 (41.3)	
Mitaka City	316 (20.3)	138 (21.9)	178 (19.2)	
Kita City	245 (15.8)	82 (13.0)	163 (17.6)	
Adachi City	158 (10.2)	61 (9.7)	97 (10.5)	
Minato City	82 (5.3)	36 (5.7)	46 (5.0)	
Machida City	66 (4.2)	21 (3.3)	45 (4.9)	
Katsushika City	19 (1.2)	5 (0.8)	14 (1.5)	
Employment status				
Currently working	1,241 (79.9)	497 (79.0)	744 (80.4)	0.49^(a)^

Abbreviation: ANOVA: analysis of variance; SD: standard deviation. *p-values for the difference in the distributions of each group were calculated using Pearson’s χ^2^ test (a) or ANOVA (b).

Table 2 presents the distribution of patient behaviors regarding follow-up visits, hospital treatments, and medical checkups or screenings during the SOE. Among men, 12.7% reported having discontinuing or postponing follow-up visits or treatments during the SOE, compared to 20.4% of women. The most commonly discontinued or postponed treatments, in descending order, were dental care, eye diseases, hypertension, cancer, and diabetes. The most frequent reason for discontinuing was “wanted to avoid contact with other people,” cited by 57.5% of men and 51.9% of women. Regarding medical checkups or screenings during the SOE, 6.5% of men and 14.2% of women stated they “decided not to take the check up,” while 3.2% men and 3.8% of women said they “canceled or postponed at the request of the hospital.” Among those who chose “canceled or postponed at the request of the hospital,” most responded “Yes” when asked if they had received a medical checkup after the SOE was lifted. Of those who chose “decided not to take the checkup,” only 24.4% of men and 36.6% of women responded "Received medical checkup” after the SOE.

**Table 2.** Patient Behaviors for Follow-Up Visits or Treatment at Hospital and Medical Checkup or Screening for the Prevention of Disease During the SOE.

Questions	Overall	Men	Women	p*
	n = 1,554	n = 629	n = 925	
**Discontinued or postponed follow-up visits or treatment during the SOE, n (%)**				p^(a)^
Yes	269 (17.3)	80 (12.7)	189 (20.4)	<0.01
No (no scheduled follow-up visits or treatment)	1,254 (80.7)	540 (85.9)	714 (77.2)	0.01
No answer	31 (2.0)	9 (1.4)	22 (2.4)	
**Disease group for follow-up visits or treatment that was discontinued or postponed during the SOE, n (%)**				
(Question only for those who postponed or canceled visits)	n = 269	n = 80	n = 189	p^(a)^
Dental care and regular checkups	82 (30.5)	14 (17.5)	68 (36.0)	<0.01
Eye disease	43 (16.0)	13 (16.3)	30 (15.9)	0.19
Hypertension	35 (13.0)	15 (18.8)	20 (10.6)	0.84
Cancer	32 (11.9)	11 (13.8)	21 (11.1)	0.4
Diabetes	15 (5.6)	11 (13.8)	4 (2.1)	<0.01
Disease of the brain or heart	13 (4.8)	4 (5.0)	9 (4.8)	0.36
Ear disease	8 (3.0)	3 (3.8)	5 (2.6)	0.86
Others	88 (32.7)	31 (38.8)	57 (30.2)	<0.01
No answer	11 (4.1)	4 (5.0)	7 (3.7)	
**Reason for discontinued or postponed follow-up visits or treatment during the SOE, n (%)**				
(Question only for those who postponed or canceled visits)	n = 269	n = 80	n = 189	p^(a)^
Wanted to avoid contact with other people	144 (53.5)	46 (57.5)	98 (51.9)	0.01
Unwilling to go to the hospital.	87 (32.3)	20 (25.0)	67 (35.4)	<0.01
Cancelled or postponed at the request of the hospital.	33 (12.3)	13 (16.3)	20 (10.6)	0.79
**Medical checkup or screening during the SOE, n (%)**	n = 1,554	n = 629	n = 925	<0.01^(b)^
Cancelled or postponed at the request of the hospital	55 (3.5)	20 (3.2)	35 (3.8)	
Decided not to take the checkup	172 (11.1)	41 (6.5)	131 (14.2)	
Did not plan to take the checkup	983 (63.3)	408 (64.9)	575 (62.2)	
Received a scheduled medical checkup	314 (20.2)	143 (22.7)	171 (18.5)	
No answer	30 (1.9)	17 (2.7)	13 (1.4)	
**Medical checkup or screening after the SOE, n (%)**	n = 1,554	n = 629	n = 925	0.83^(b)^
Yes: Received medical checkup	1,040 (66.9)	412 (65.5)	628 (67.9)	
No: Did not receive a medical checkup	360 (23.2)	152 (24.2)	208 (22.5)	
Scheduled to receive a medical checkup but have not yet	115 (7.4)	43 (6.8)	72 (7.8)	
No answer	39(2.5)	22(3.5)	17(1.8)	
**Medical checkup or screening after the SOE**				
(Question only for those who canceled a medical checkup or screening)	n = 55	n = 20	n = 35	0.04^(b)^
Yes: Received medical checkup	45 (81.8)	14 (70.0)	31 (88.6)	
No: Did not receive a medical checkup	3 (5.5)	1 (5.0)	2 (5.7)	
Scheduled to receive a medical checkup but have not yet	5 (9.1)	3 (15.0)	2 (5.7)	
No answer	2 (3.6)	2 (10.0)	0 (0.0)	
**Medical checkup or screening after the SOE**				
(Question only for those who decided not to receive a medical checkup or screening. )	n = 172	n = 41	n = 131	0.12^(b)^
Yes: Received medical checkup	58 (33.7)	10 (24.4)	48 (36.6)	
No: Did not receive a medical checkup	82 (47.7)	19 (46.3)	63 (48.1)	
Scheduled to receive a medical checkup but have not yet	28 (16.3)	9 (22.0)	19 (14.5)	
No answer	4 (2.3)	3 (7.3)	1 (0.8)	

Abbreviation: SOE, state of emergency. *p-values for the difference in the distributions of each group were calculated using Pearson’s χ^2^ test (a) or ANOVA (b).

Table 3 shows the associations between patient behaviors and changes in work, income, and lifestyle during the SOE. Over 40% of both men and women reported work changes (e.g., changes in hours, increased remote work, wage changes), and 22.7% of men and 24.6% of women reported a decrease in household income. No relationship was found between these factors and medical examination behavior. The association between housework and family care time increased for 37.7% of women (21.9% of men) and was statistically significant only in women, particularly in relation to refraining from medical examinations (odds ratio [OR] 1.55; 95% confidence interval [CI]: 1.08-2.23). Among those who reported increased stress or irritability with roommates, 31.8% of men and 8.9% of women reported this, with a statistically significant association with refraining from seeing a doctor observed only in women (OR 1.53; 95% CI: 1.06-2.21). Regarding lifestyle changes, few men and women reported increased smoking, but men who reported increased alcohol consumption were significantly more likely to have discontinued or postponed follow-up visits or treatment (OR 1.46 95% CI: 1.00-2.13). Approximately 30% of men and over 40% of women reported weight gain, but only women who reported increased food intake were significantly associated with discontinued or postponed follow-up visits or treatment (OR 1.46 95% CI: 1.00-2.13).

**Table 3.** Associations of Patient Behavior with Discontinued or Postponed Follow-Up Visits or Treatment for Changes in Work, Income, and Lifestyle During the SOE.

Questions			Men (n = 629)					Women (n = 925)			
			Model 1		Model 2			Model 1		Model 2	
		n (%)	OR	95% CI	OR	95% CI	n (%)	OR	95% CI	OR	95% CI
**Some major changes in work** (i.e., working hours, environment, and payment)	Yes	250 (49.0)	1.31	(0.77-2.23)	1.78	(0.87-3.66)	297 (42.4)	1.37	(0.94-2.00)	1.30	(0.84-2.03)
	No		1.00		1.00			1.00		1.00	
**Decreased household income**	Yes	143 (22.7)	1.01	(0.57-1.78)	1.02	(0.57-1.83)	228 (24.6)	0.99	(0.69-1.45)	0.99	(0.68-1.46)
	No		1.00		1.00			1.00		1.00	
**Increased household time and/or family care**	Yes	138 (21.9)	1.19	(0.60-2.38)	1.18	(0.59-2.39)	349 (37.7)	1.39	(0.98-1.97)	1.55	(1.08-2.23)
	No		1.00		1.00			1.00		1.00	
**Increased stress, arguments, and irritability with household members**	Yes	193 (31.7)	1.37	(0.80-2.35)	1.33	(0.78-2.29)	431 (48.9)	1.51	(1.05-2.17)	1.53	(1.06-2.21)
	No		1.00		1.00			1.00		1.00	
**Change in lifestyle**		
Increased smoking	Yes	12 (1.9)	2.31	(0.60-8.86)	2.23	(0.57-8.64)	11 (1.2)	1.35	(0.35-5.16)	1.13	(0.29-4.38)
	No		1.00		1.00			1.00		1.00	
Increased alcohol consumption	Yes	72 (11.4)	1.95	(0.99-3.86)	2.05	(1.03-4.10)	72 (7.8)	1.42	(0.81-2.51)	1.52	(0.85-2.71)
	No		1.00		1.00			1.00		1.00
Body weight gain	Yes	188 (29.9)	1.36	(0.82-2.25)	1.40	(0.84-2.34)	380 (41.1)	1.04	(0.74-1.46)	1.06	(0.75-1.50)
	No		1.00		1.00			1.00		1.00	
Increased food intake	Yes	64 (10.2)	1.85	(0.95-3.60)	1.87	(0.96-3.65)	212 (22.9)	1.43	(0.98-2.07)	1.46	(1.00-2.13)
	No		1.00		1.00			1.00		1.00	
Increased physical activity	Yes	78 (12.4)	1.47	(0.71-3.07)	1.43	(0.68-2.98)	118 (12.8)	0.73	(0.42-2.07)	0.71	(0.41-1.23)
	No		1.00		1.00			1.00		1.00	

Model 1: crude model Model 2: adjusted for gender, age (category), area and employment status Abbreviations: CI, confidence interval; OR, odds ratio; SOE, state of emergency.

Table 4 presents the association between patient behavior and questionnaire scores. On the UCLA Three-item loneliness scale, 20.1% of men and 22.9% of women scored six or higher, indicating they were “lonely.” On the K6 scale, 18.6% of men and 29.3% of women scored between five and 12 points, indicating mild psychological distress, while 4.2% of men and 3.1% of women scored 13 or higher, indicating severe psychological distress. On the FCV-19S scale, 11.7% of men and 13.6% of women scored 21 or higher, though the threshold for this scale has not been established. Logistic regression analysis, comparing the low-score group of each scale to the refraining-from-seeing-a-doctor, showed the following ORs: 1.22 (95% CI: 0.88-1.68) for the UCLA Three-item loneliness scale; 1.32 (95% CI: 0.98-1.78) and 1.18 (95% CI: 0.64-2.17) for K6; and 1.31 (95% CI: 0.93-1.86) and 1.24 (95% CI: 0.77-1.99) for FCV-19S. None of these differences were statistically significant after adjusting for covariates, and the results were consistent when the analyses were stratified by gender.

**Table 4.** Associations of Patient Behavior with Discontinued or Postponed Follow-Up Visits or Treatment for Scores on Mental Health Questionnaires During the SOE.

Mental Health Questionnaires		Overall	Men	Women	Model 1		Model 2	
		n = 1,523	n = 618	n = 905	OR	95% CI	OR	95% CI
**UCLA Loneliness Scale, n (%)**								
score	**3-5**	1,192 (78.3)	494 (79.9)	698 (77.1)	1.00			
	**6-9**	331 (21.7)	124 (20.1)	207 (22.9)	1.17	(0.86-1.61)	1.22	(0.88-1.68)
**K6, n (%)**								
Score	**0-4**	1,079 (72.1)	484 (78.3)	595 (65.8)	1.00			
	**5-12**	380 (30.0)	115 (18.6)	265 (29.3)	1.41	(1.05-1.89)	1.32	(0.98-1.78)
	**13-24**	64 (4.2)	19 (3.1)	45 (5.0)	1.22	(0.67-2.24)	1.18	(0.64-2.17)
**FCV-19S, n (%)**								
score	**7-10**	352 (23.1)	181 (29.3)	171 (18.9)	1.00			
	**11-20**	976 (64.1)	365 (59.1)	611 (67.5)	1.44	(1.02-2.02)	1.31	(0.93-1.86)
	**21-35**	195 (12.8)	72 (11.7)	123 (13.6)	1.41	(0.88-2.24)	1.24	(0.77-1.99)

Model 1: crude model Model 2: adjusted for gender, age (category), area and employment status Abbreviations: CI, confidence interval; FCV-19S, Fear of Coronavirus-19 Scale; K6, Kessler 6 scale; OR, odds ratio; SOE, state of emergency; UCLA, University of California, Los Angeles

## Discussion

We conducted a paper-based questionnaire survey to assess patient behavior in Tokyo during the COVID-19 pandemic. We found that 12.7% of men and 20.4% of women postponed or discontinued follow-up visits or treatment during the SOE from April to May 2020. Regarding medical checkups or disease prevention screening, 6.5% of men and 14.2% of women stopped these activities during the SOE, with only half indicating an intention to resume or plan to resume them after the SOE was lifted. Several similar studies in Japan ^(5), (6)^ have also reported that some patients avoided healthcare facilities to prevent infection and expressed intentions to continue this behavior even after COVID-19 was under control.

According to the Ministry of Health, Labour, and Welfare (MHLW) ^(15)^, the average daily hospital outpatient volume in 2020 was 9.9% lower than in 2019, but it increased by 4.2% in 2021 compared to 2020. A research group from MHLW ^(16)^ reported that the number of people undergoing cancer screening in 2020 was about 10% lower than in 2019. As concerns about COVID-19 infection decrease, the use of healthcare services is expected to recover. However, there are concerns that the “pandemic behavior” may become habitual, with some patients continuing to avoid healthcare services. On the positive side of pandemic-related behavioral changes, patients with minor illnesses, such as colds, stopped visiting hospitals. Infection prevention measures, including handwashing, gargling, and wearing masks, likely reduced other infections. In fact, a significant decrease in diseases transmitted by droplet and contact infection was observed in Japan in 2020 ^(17)^, with a consequent reduction in unnecessary medical visits ^(18), (19)^. In Japan, the rapidly aging society and the rising cost of healthcare are ongoing concerns. Several measures have been implemented to alleviate these issues, such as preventing lifestyle-related diseases and reducing the average length of hospital stays ^(20)^. It is important to note that healthcare utilization has decreased more than expected based on typical seasonal variation, even for conditions requiring urgent care, such as acute coronary syndrome ^(21), (22)^. Although this study does not clarify whether the decrease in visits was due to chronic or acute illnesses, the reduction in emergency care access is concerning. Unlike chronic diseases, emerging infectious diseases require measures to prevent the spread of infection and prompt response at the onset of illness. Additionally, since the prognosis of infectious diseases depends on the status of the underlying chronic conditions, regular checkups and examinations are critical. Communicating the importance of ongoing treatment through patient education and disseminating evidence-based information on patient behavior during pandemics is essential. Effective communication will require cooperation among medical providers, government bodies, and local governments.

Several other findings from our survey merit discussion. Regarding work and income, about 20% of participants reported decreases in both compared to their pre-pandemic situations. In a June 2020 survey by the Bank of Japan ^(23)^, 39.1% of respondents reported a decrease in income, a figure that remained unchanged in the Bank’s September 2022 survey ^(24)^. Despite stable unemployment rates ^(25)^, payroll reductions were widespread. The reasons for these declines likely vary by industry and type of employment, although we did not collect such data. Given the potential impact of socioeconomic status on patient behavior during a SOE, it is necessary to collaborate with government and local municipalities to gather evidence and ensure that critical information reaches the patients. In terms of lifestyle factors, there was a trend toward less healthy behaviors, particularly among women, in relation to body weight, exercise, and food intake. However, it is not possible to conclude overall health status declined, as precise measurements of weight and food intake were not measured. In this group, stress may have been a contributing factor, as more than 40% of women and 20% of men reported eating their favorite foods to cope with stress. Regarding mental health, 20.1% of men and 22.9% of women felt lonely, and 2.9% and 3.7%, respectively, experienced severe psychological distress during the pandemic. However, there was no statistically significant association between these factors and discontinued or postponed follow-up visits or treatment. These findings differ from other studies ^(26), (27), (28)^ that reported higher levels of loneliness and distress, likely due to differences in participant age, region, and study period.

Our survey revealed multiple aspects of patient behavior in Tokyo during the COVID-19 pandemic. One of the strengths of our study is that the survey framework was already in place for urban residents at the time the pandemic began. Epidemiological surveys targeting large populations, especially in urban areas with diverse lifestyles and frequent population movement, require significant time and effort for follow-up, such as in setting up and recruiting participants. In our study, this process was facilitated by ethical screening and the consent collection during the annual survey. However, several limitations must be considered. First, the sample was not adjusted to represent the general Tokyo population, as all participants had undergone gastric cancer screening. In Japan, the target population for gastric cancer screening is individuals over 40 years old, meaning more than 99% of our participants were over 40. Previous research has shown that younger individuals were more likely to experience psychological distress and had difficulty maintaining regular medication use during the pandemic ^(6)^, suggesting that our study may underestimate the psychological impact on younger people. Second, our study cohort consisted of individuals who had undergone cancer screening, and cancer screening participants are likely more health conscious and have healthier lifestyles than the general population. As a result, our findings may not fully reflect the experiences of those who are less-health conscious or who do not participate in cancer screening. Additionally, our results may not accurately capture the impact on lower-income individuals, who were more reported to experience greater psychological distress during the pandemic ^(27)^ and were less likely to undergo cancer screening ^(29)^. Our study did not gather information on participants’ socioeconomic status, limiting the analysis. Third, the cross-sectional nature of this study makes it difficult to establish causality, as we cannot definitively attribute behavioral changes to COVID-19. Finally, data were collected 1 to 2 years after the SOE was declared, which may have introduced recall bias.

### Conclusions

This survey revealed that many middle-aged and older Japanese refrained from using medical services to avoid COVID-19 infection, and some continued this behavior even after the pandemic subsided. To ensure the proper management of chronic diseases and prevent further health issues, patients should be educated about the importance of regular medical examinations and checkups. Furthermore, methods for disseminating information in response to this trend should be developed in collaboration with national and local governments.

## Article Information

### Acknowledgments

We are grateful to all participants and staff members for their support in the conduct of this survey.

### Author Contributions

Concept and design of the study and supervision of the project: Manami Inoue. Planning and conduction of the study: Natsumi Terada, Ayami Ono, Shiori Tanaka and Tetsuji Minami. Analysis of the data: Natsumi Terada and Ayami Ono. Writing of the manuscript: Natsumi Terada. Interpretation of the data and critical revision the paper: Manami Inoue, Natsumi Terada, Ayami Ono, Shiori Tanaka and Tetsuji Minami. Reanalyzing and revising the paper: Ayami Ono and Manami Inoue. Recruitment of research participants in the study area: Fuyuki Yamada, Toshiki Yamashita, Sawori Osada, Hitoshi Mio, Masahiko Nakamura, Masakatsu Uchihara, Nakayuki Yoshimura, Yasuhiko Nagata, Mikio Masuda, Ryohei Itagaki, Akira Torii and Toru Kakuta. All authors reviewed the manuscript and contributed to its revision. Natsumi Terada and Ayami Ono equally contributed to the manuscript and are recognized as co-first authors.

### Conflicts of Interest

None

### Disclaimer

Toru Kakuta is one of the JMA Officers in Charge of JMA Journal. He was not involved in the editorial evaluation or decision to accept this article for publication at all.
